# 1400. Geospatial Modeling of the Prevalence of Pediatric Febrile Illness in Uganda

**DOI:** 10.1093/ofid/ofad500.1237

**Published:** 2023-11-27

**Authors:** Paddy Ssentongo, Khush Shah

**Affiliations:** Penn State Hershey Med Center, Hershey, Pennsylvania; Millersville University, Millersville, Pennsylvania

## Abstract

**Background:**

Febrile illness is still one of the major public health problems in Africa. Although it has been recognised as crucial to identify the non-malarial aetiologies and to better understand their spatial distribution, the evidence is still limited. We aim to predict the prevalence of fever cases among children aged under 5 years in Uganda using geostatistical models, whilst exploring the potential association with environmental, nutritional, and socio-demographic factors.

**Methods:**

We analyze household records from the 2016 Demographic and Health Surveys (DHS) conducted in Uganda. We develop a geostatistical Binomial model to predict the prevalence of fever cases among children aged under 5 years in Uganda, accounting for environmental, nutritional, and socio-demographic risk factors obtained from publicly available sources.

**Results:**

The average number of children in each cluster was 20 (interquartile range: 16–25), and the median crude (empirical) febrile illness prevalence for each cluster was 30.4% (interquartile range:13.6–50.0). The model indicates the presence of a strong spatial correlation, predicting a higher prevalence in the eastern and north-eastern regions. The inclusion of some potential risk factors improves the predictive performance of the model. Key environmental, nutritional, and socio-demographic factors strongly associated with febrile illness included

**Conclusion:**

We show that there is extensive within-country spatial variation in children’s febrile illness prevalence in Uganda, whilst indicating the potential association with rainfall, temperature, aridity, poverty, standardized precipitation evapotranspiration index, and anemia. These findings could assist in targeted public health policymaking for fever case management and in hypothesis generation for etiology.

**Disclosures:**

**All Authors**: No reported disclosures

